# Three-Dimensional Deformations of Pulmonary Collapse for Intraoperative Augmented Reality Guidance: A Proof-of-Concept Study

**DOI:** 10.1177/15569845251401314

**Published:** 2026-02-01

**Authors:** Jette J. Peek, Tjerko Kieft, Rahi S. Alipour Symakani, Amir H. Sadeghi, Mathieu M.E. Wijffels, Esther M.M. Van Lieshout, Ad J.J.C. Bogers, Edris A.F. Mahtab

**Affiliations:** 1 Department of Cardiothoracic Surgery, Erasmus MC, University Medical Center Rotterdam, The Netherlands; 2 Surgical Reality, Nieuw-Vennep, The Netherlands; 3 Department of Cardiology, Division of Experimental Cardiology, Erasmus MC, University Medical Center Rotterdam, The Netherlands; 4 Department of Pediatrics, Division of Pediatric Cardiology, Erasmus MC, University Medical Center Rotterdam, The Netherlands; 5 Department of Cardiothoracic Surgery, University Medical Center Utrecht, The Netherlands; 6 Trauma Research Unit Department of Surgery, Erasmus MC, University Medical Center Rotterdam, The Netherlands; 7 Department of Cardiothoracic Surgery, Leiden University Medical Center, The Netherlands

**Keywords:** pulmonary collapse, bronchial deformation, pulmonary surgery, pneumothorax, augmented reality, intraoperative guidance, deformation modeling

## Abstract

**Objective:**

During pulmonary surgery, the lung is deflated to facilitate the procedure. This study aimed to assess the deformation of the bronchial tree and pulmonary parenchyma during lung collapse, for eventual use in augmented reality (AR) guidance during pulmonary resections.

**Methods:**

The concept was first tested in 2 porcine models by analyzing paired computed tomography scans of collapsed and inflated lungs, then applied to 6 human patients. Bronchus and parenchyma were segmented, and a bronchus centerline was calculated. The diameter, length differences, angular deformations, and volume differences of the parenchyma were calculated. Finally, these deformations were applied on the inflated bronchus centerline to generate an artificially collapsed bronchus.

**Results:**

In both the porcine and human models, the pulmonary collapse resulted in substantial volumetric and anatomical changes. For the humans, the right lung showed a median displacement of 14.41 mm in the dorsomedial direction, while the left lung was displaced 11.99 mm in the dorsolateral direction (*P* = 0.79). Median volume reduction was 970 mL for the right lung and 878 mL for the left lung. Bronchial narrowing was observed, with a median diameter reduction of 0.14 mm for the right lung and 1.23 mm for the left lung. Moreover, the lengths of the bronchial segments were reduced, with a median length reduction of 0.20 mm for the right sided and 0.72 mm for the left sided.

**Conclusions:**

Algorithmically driven calculations of the intraoperative pulmonary collapse of human and porcine lungs were performed and applied onto an inspirated bronchus. This resulted in an artificial collapsed bronchus. This method could be a foundation for a dynamical deformable deflation model, suitable for intraoperative AR-based pulmonary navigation.

## Graphical abstract

**Visual abstract:**
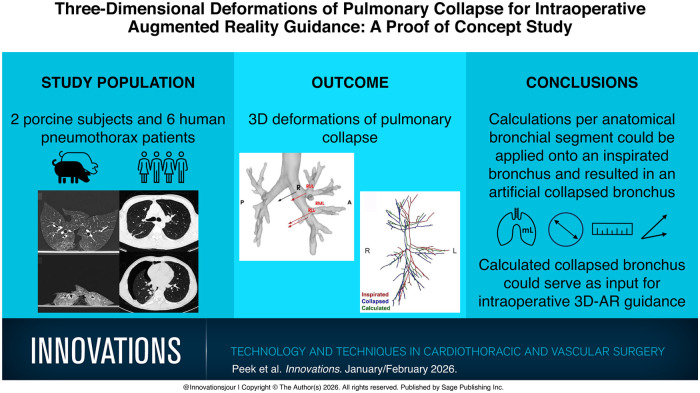

**Central Message**

We performed an algorithmic analysis of deformations during lung collapse from porcine lungs and human pneumothorax patients. These calculated deformations were applied to an inflated bronchus using a mathematical algorithm, generating an artificial collapsed bronchus. This method serves as a foundation for developing accurate deflation models improving intraoperative pulmonary AR guidance.

## Introduction

Preoperative 3-dimensional (3D) reconstructions are highly recommended and widely used in pulmonary segmentectomy, for tumor localization and recognition of bronchovascular anatomical variations.^1^ These 3D reconstructions are based on preoperative computed tomography (CT) scans where the lungs are ventilated. However, during pulmonary surgery, the lung is deflated to facilitate the procedure, and these 3D reconstructions differ significantly from the intraoperative deflated state of the lungs.^1^ Beyond the deflations, the lungs are additionally distorted due to the surgical manipulations. Consequently, the intraoperative anatomy, orientation, and spatial relationships are significantly different compared with the preoperative CT scans as well as the 3D reconstructions.^1,2^ Understanding this altered anatomy can be challenging, even for experienced surgeons. This is particularly true during complex resections such as a segmentectomy, in which precise identification of smaller bronchovascular branches is important.^3^ To account for these challenges, several pulmonary simulation models have been proposed in the literature, providing a dynamic, more accurate representation of the intraoperative altered anatomy.^3–5^ Augmented reality (AR) is proposed for intraoperative application of preoperative 3D models for achieving safer, more accurate surgical resections.^4^ With intraoperative AR, we refer to an overlay of a preoperative 3D model onto the surgical field, aiding the surgeon by identifying the intraoperative anatomy. The accuracy of these 3D-AR overlays highly depends on representative preoperative 3D models.

Since intraoperative cone-beam CT (CBCT) imaging of the lungs is not routinely performed in most centers, we studied posttraumatic pneumothorax patients to obtain data on deflated lungs. Pneumothorax is a clinical condition in patients mimicking the deflation of lungs, outside the operating room. To improve the accuracy of simulated pulmonary models, deflation of the lung needs to be incorporated into these 3D-AR models. Several methods have been described for assessing pneumothorax deformation, such as kernel-based modeling, based on anatomical landmarks and model-based registration for calculating the deformation in vivo in Beagle dogs.^6–8^ The mechanism of pulmonary deflation is studied mostly in animals but is still complex and findings are incomplete.^9^

In this experimental study, we aimed to assess the deformation of the bronchial tree and the parenchymal volume changes during pulmonary collapse. Computational analysis of artificial intelligence (AI)–based 3D segmentations of CT scans was conducted to assess the pulmonary deformations. We first analyzed paired CT scans of pneumothorax and fully inspirated lungs in isolated porcine lungs for feasibility, followed by an analysis of pneumothorax in human patients.

## Methods

### Data Collection

The research protocol was approved by the Erasmus MC Medical Research Ethics Committee with reference number MEC-2023-0080. Moreover, the use of porcine lungs was approved by the Erasmus MC Institute for Animal Welfare with reference number 2216367. Informed consent was waived for patients identified from the RIB-AI study (MEC-2023-0039) because of bias due to incomplete information and high mortality in their clinical course.

### Porcine Lungs

**Fig. 1.**
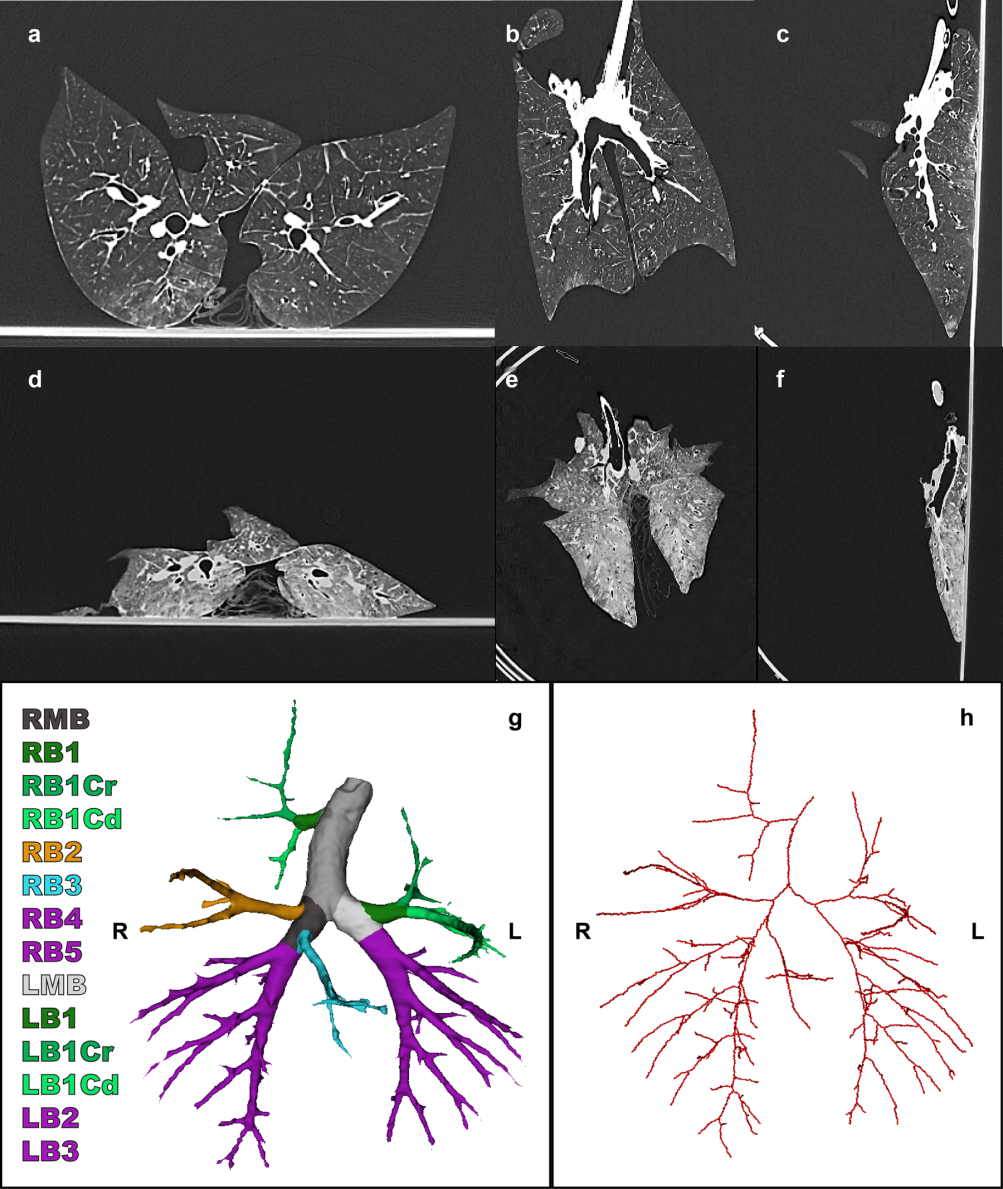
(a) Axial, (b) coronal, and (c) sagittal views of porcine CT scan in the inspirated state. (d) Axial, (e) coronal, and (f) sagittal views of porcine collapsed CT scan of the same subject. (g) Bronchus segmentation and (h) bronchus centerline. CT, computed tomography; LB1, left upper lobe; LB1Cr, cranial segment left upper lobe; LB1Cd, caudal segment left upper lobe; LB2, left lower lobe cranial; LB3, left lower lobe caudal; LMB, left main bronchus, RB1, right upper lobe; RB1Cr, cranial segment right upper lobe; RB1Cd, caudal segment right upper lobe; RB2, right middle lobe; RB3, right accessory lobe; RB4, right lower lobe cranial; RB5, right lower lobe caudal; RMB, right main bronchus.

Porcine lungs from female Yorkshire/Landrace pigs of 47 kg (Roefs, Woensdrecht, The Netherlands) were explanted en bloc as described in the literature.^10^ After retrieval, the left atrium, pulmonary artery, and trachea were cannulated for contrast administration and ventilation, as described for performing ex vivo lung perfusion.^11^ After retrieval, dual-source photon-counting detector CT (PCD-CT; Siemens NAEOTOM Alpha, Siemens Healthineers VA50, Erlangen, Germany) scans were acquired. These PCD-CT scans were acquired in the inflated state with positive end-expiratory pressure of 9 cmH_2_O and an inspirational pressure of 35 cmH_2_O. In addition, a PCD-CT was acquired in the deflated state, during apnea by disconnecting the ventilation. An example of the porcine CT scans is shown in Figure 1a–f.

### Human Lungs

**Fig. 2.**
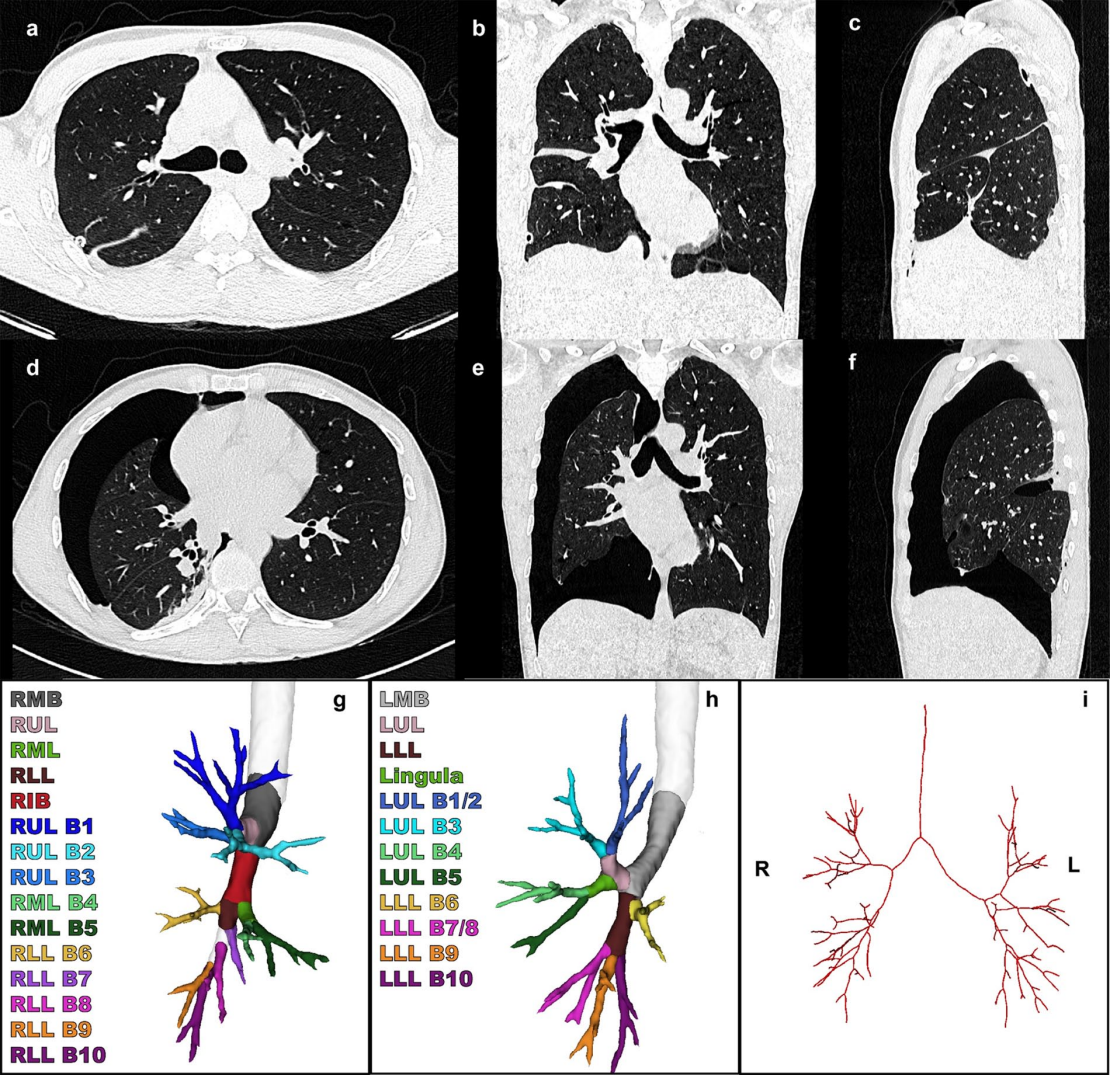
(a) Axial, (b) coronal, and (c) sagittal views of human CT scan in the inspirated state. (d) Axial, (e) coronal, and (f) sagittal views of human collapsed CT scan of the same subject. (g) (h) Bronchus segmentation and (i) bronchus centerline. CT, computed tomography; LLL, left lower lobe; LLL B6, superior segment; LLL B7/8, anterior medial basal segment; LLL B9, lateral basal segment; LLL B10, posterior basal segment; LMB, left main bronchus; LUL, left upper lobe; LUL B1/2, apicoposterior segment; LUL B3, anterior segment; LUL B4, superior lingular segment; LUL B5, inferior lingular segment; RIB, right intermediate bronchus; RLL, right lower lobe; RLL B6, superior segment; RLL B7, medial basal segment; RLL B8, anterior segment; RLL B9, lateral basal segment; RLL B10, posterior basal segment; RMB, right main bronchus; RML, right middle lobe; RML B4, lateral segment; RML B5, medial segment; RUL, right upper lobe; RUL B1, apical segment; RUL B2, posterior segment; RUL B3, anterior segment.

Patients with traumatic, total, single-sided pneumothorax with (at least) 2 available thorax CT scans were identified from the database of the RIB-AI study (MEC-2023-0039). Informed consent was waived for these patients because of bias due to incomplete information and high mortality in their clinical course. Patients with a unilateral fully collapsed lung were included, with the lobes being collapsed at the cranial, dorsal, lateral, and ventral sides. Patients were excluded if no reference CT scan was available, if no full pneumothorax was apparent, or if they had visible structural pulmonary abnormalities including large hemothorax, large lobar hematomas, or open chest wall wounds due to their severe trauma. Examples of the human CT scans are shown in Figure 2a–f.

### Segmentation

The 3D segmentation of CT scans was performed of the parenchyma and the bronchus from both porcine and human lungs. The parenchyma was segmented using 3D Slicer (3D Slicer Community, Boston, MA, USA) with the TotalSegmentator extension, and deep learning–based segmentation was performed of the bronchial tree.^4,12,13^ After segmentation of the pulmonary parenchyma and the bronchial tree, Python 3.11.9 (Python Software Foundation, Wilmington, DE, USA) was used to generate centerlines of the bronchial tree segmentation, adapting sknw and SimpleITK packages. Bronchial segmentations are visualized in Figure 1g and Figure 2g–h and the centerlines in Figure 1h and Figure 2i for the porcine and human studies, respectively.

### Outcomes

Because the focus was specifically on bronchial displacement, we performed direct registration of the bronchus centerlines. For this, we used the noncollapsed bronchus side as a reference, as these branches remain unaffected by the collapse. We registered the centerlines based on the location of the main carina and the contralateral main bronchus to be able to adequately compare the collapsed side of the bronchial tree. After registration of the centerlines, we labeled the segmental branches of the bronchial tree to compare the individual segments. These labels are shown in Figure 1g and Figure 2g–h for porcine and human studies, respectively. After labeling the segments accordingly, we converted the branches to a straight vector using a linear least square approximation of all the points of the branch, to enable calculation of the displacement vector between the bronchial segments. To calculate the vector between 2 corresponding segments (for example, between the right main bronchus [pneumothorax] with the right main bronchus [nonpneumothorax]):

$$\vec{d_{b}}=\vec{c_{b}}-\vec{\iota_{b}},$$

with the absolute difference $\vec{d_{b}}$ of branch b between the collapsed, pneumothorax vector $\vec{c_{b}}$ and the inspirated, nonpneumothorax vector $\vec{\iota_{b}}$ of branch b . The angle between 2 branches $\theta_{v}$ is calculated from the vectors between the branches:

$$\theta_{v}=\tan^{-1}\left(\frac{v_{x}}{v_{y}}\right)$$

where the x and y component are corresponding to the specific view: axial(x,y), sagittal(x,z), or coronal(y,z).

The deformations of a branch were calculated independently, by subtracting deformations of the branches between the trachea and given branch. This ensures that the deformation calculated for a branch is explicitly for that specific branch. For example, a rotational deformation of the right upper lobe bronchus has a direct effect on all segmental branches of the right upper lobe. Subtracting these deformations accordingly resulted in independent deformation calculations per individual branch. Displacement vector length was calculated to represent the amount of displacement in millimeters.

Moreover, the lengths of all separate centerline branches were calculated by the Euclidean distance between all points of the branch. This way, the shape of the bronchial branch was taken into account, instead of calculating the absolute distance between the 2 endpoints of the branch.

The maximum diameter was calculated from all separate branches, by calculating the orthogonal distance from the centerline to the boundary of the bronchus segmentation. This was performed in multiple directions and therefore gave an accurate approximation of the surface shape of the cross section of the bronchus.

Parenchymal volume was calculated for the human lungs per lobe, whereas the total lung volume was calculated in the porcine lung models.

Finally, after calculating the deformations, we applied these deformations (length and angular differences) per branch onto the branches of the inflated bronchus centerline to generate an algorithmically modeled collapsed centerline.

### Statistical Analysis

Because only 2 pairs of porcine lung CT scans were included in this study, we did not perform any statistical analysis to compare human and porcine lung collapse. We compared the collapsed and inflated human lungs per branch using the Wilcoxon signed rank test using IBM SPSS Statistics, Version 28.0.1.0 (IBM Corp, Armonk, NY, USA).

## Results

Two porcine subjects were included in this analysis. We screened 374 patients, of whom 32 patients had 2 or more available CT scans. Of these patients with multiple CT scans, we excluded 26 patients because of incomplete pneumothorax (*n* = 20), structural pulmonary abnormalities because of trauma (*n* = 4), or insufficient image quality for analysis (*n* = 2), and we included 6 patients with a full unilateral pneumothorax. Of the included patients, 3 patients had a right-sided pneumothorax, and 3 had a left-sided pneumothorax. Two patients had a drain in situ and 1 was on mechanical ventilation. The bronchus and the pulmonary parenchyma were segmented successfully from all human and porcine CT scans, and a bronchus centerline was created for calculating the length, diameter, and displacement of all bronchial tree segments.

### Lung Volume

**Fig. 3.**
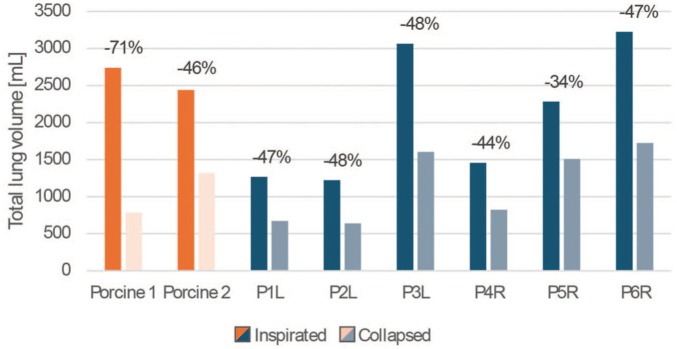
Volume differences between inflated and collapsed lungs for the patients with L and R pneumothorax and both porcine lungs. L, left sided; R, right sided.

In the porcine lungs, the parenchymal volume decreased by 1,957.3 mL and 1,123.5 mL between the inflated and collapsed states, respectively, for both cases. In the human cohort, the average parenchymal volume decrease was 970.6 mL for the right-sided pneumothorax and 878.8 mL for left-sided pneumothorax cases (*P* = 0.109). In Figure 3, the volume differences are shown for all subjects in a graph.

### Diameter and Length Differences

The diameters and lengths of all individual bronchial segments were calculated. We observed narrowing and shortening of the bronchial trees. In the porcine lungs, the median diameter reduction was 1.30 mm for all bronchial branches, and the median length reduction was 1.26 mm between the inspirated and the collapsed bronchi (Table 1).

**Table 1.** Diameter and Length of Porcine Bronchial Tree Segments.

	Porcine 1			Porcine 2		
	Inspirated, mm	Collapsed, mm	Difference, mm (%)	Inspirated, mm	Collapsed, mm	Difference, mm (%)
*Diameter*						
Trachea	16.85	13.84	−3.01 (−17.9%)	16.84	14.82	−2 (−12%)
Left						
LMB	11.09	9.64	−1.45 (−13.0%)	12.10	10.38	−1.7 (−14%)
LB1	5.47	4.17	−1.30 (−23.7%)	5.56	3.67	−1.9 (−34%)
LB1Cr	2.29	2.11	−0.18 (−7.8%)	2.94	2.02	−0.9 (−31%)
LB1Cd	4.57	4.04	−0.53 (−11.6%)	3.65	2.35	−1.3 (−36%)
LB2	8.34	6.19	−2.15 (−25.8%)	9.02	7.39	−1.6 (−18%)
LB3	5.60	3.02	−2.58 (−46.1%)	3.34	3.19	−0.2 (−5%)
Right						
RMB	14.12	10.84	−3.28 (−23.2%)	13.00	10.43	−2.6 (−20%)
RB1	4.79	2.74	−2.05 (−42.8%)	4.19	3.20	−1 (−24%)
RB1Cr	3.38	2.37	−1.01 (−29.9%)	3.33	3.25	−0.1 (−2%)
RB1Cd	2.98	2.95	−0.03 (−1.0%)	3.34	1.57	−1.8 (−53%)
RB2	4.65	3.97	−0.68 (−14.6%)	4.45	3.83	−0.6 (−14%)
RB3	3.17	2.59	−0.58 (−18.4%)	3.05	2.72	−0.3 (−11%)
RB4	10.31	7.12	−3.19 (−31.0%)	9.61	7.34	−2.3 (−24%)
RB5	2.64	1.98	−0.66 (−24.8%)	2.78	2.20	−0.6 (−21%)
*Length*						
Trachea	35.45	29.58	−5.87 (−17%)	37.00	35.77	−1.23 (−3.3%)
Left						
LMB	19.06	16.18	−2.88 (−15%)	20.51	18.03	−2.48 (−12.1%)
LB1	18.98	17.47	−1.52 (−8%)	16.11	17.52	1.41 (8.8%)
LB1Cr	10.21	8.90	−1.30 (−13%)	9.78	9.30	−0.48 (−4.9%)
LB1Cd	13.89	13.57	−0.32 (−2%)	71.24	58.22	−13.01 (−18.3%)
LB2	23.04	18.47	−4.57 (−20%)	18.53	20.99	2.46 (13.3%)
LB3	50.62	30.70	−19.91 (−39%)	28.15	43.06	14.91 (53.0%)
Right						
RMB	11.60	11.29	−0.31 (−3%)	13.91	14.04	0.13 (0.9%)
RB1	14.54	13.53	−1.01 (−7%)	13.37	15.77	2.40 (18.0%)
RB1Cr	9.73	7.37	−2.36 (−24%)	10.09	7.48	−2.62 (−25.9%)
RB1Cd	21.75	13.63	−8.12 (−37%)	32.99	9.98	−23.02 (−69.8%)
RB2	19.54	18.23	−1.30 (−7%)	16.81	16.36	−0.45 (−2.7%)
RB3	31.57	22.77	−8.80 (−28%)	16.67	18.16	1.49 (9.0%)
RB4	15.30	14.64	−0.66 (−4%)	15.16	16.80	1.64 (10.8%)
RB5	26.66	21.97	−4.69 (−18%)	18.67	60.60	41.92 (224.5%)

Abbreviations: LB1, left upper lobe; LB1Cr, cranial segment left upper lobe; LB1Cd, caudal segment left upper lobe; LB2, left lower lobe cranial; LB3, left lower lobe caudal; LMB, left main bronchus, RB1, right upper lobe; RB1Cr, cranial segment right upper lobe; RB1Cd, caudal segment right upper lobe; RB2, right middle lobe; RB3, right accessory lobe; RB4, right lower lobe cranial; RB5, right lower lobe caudal; RMB, right main bronchus.

Negative values indicate a decrease, and positive values indicate an increase of the diameter and length.

For the human subjects, the median diameter decrease between inspirated and collapsed state was 0.14 mm for the right lung and for the left lung the median diameter decrease was 1.23 mm (Table 2). The median length reduction was 0.20 mm for the right lung and 0.72 mm for the left lung (Table 3). No statistically significant differences were found for the diameter and length comparison.

**Table 2.** Diameter of Bronchial Segments Until the Segmental Level of Human Lungs in Patients With Left and Right Pneumothorax.

	Median from inspirated CT scan, mm	Median from collapsed CT scan, mm	Diameter difference, mm (%)	Confidence interval	*P* value
Left pneumothorax					
LMB	15.37	14.63	1.14 (7.4)	−2.16 to 4.44	0.285
LUL	11.29	10.87	−1.86 (−16.5)	−2.49 to −1.23	0.285
LLL	12.47	9.61	−1.65 (−13.2)	−3.88 to 0.58	0.285
Lingula	6.67	7.17	−0.63 (−9.5)	−2.48 to 1.21	0.593
LUL B1/2	4.19	3.63	0.07 (1.8)	−1.06 to 1.20	1.000
LUL B3	3.58	4.01	0.62 (17.2)	−2.16 to 3.40	0.593
LUL B4	3.00	3.00	0.13 (4.3)	−0.16 to 0.42	1.000
LUL B5	3.63	2.93	−0.27 (−7.3)	−1.72 to 1.18	0.593
LLL B6	4.82	4.11	0.28 (5.8)	−2.76 to 3.31	1.000
LLL B7/8	4.01	3.72	−0.16 (−4.0)	−1.23 to 0.91	0.593
LLL B9	4.49	3.93	−0.46 (−10.3)	−1.37 to 0.44	0.285
LLL B10	3.46	3.63	−0.12 (−3.5)	−1.85 to 1.61	1.000
Right pneumothorax					
RMB	22.36	18.36	−1.45 (−6.5)	−4.18 to 1.27	0.285
RUL	13.67	11.77	−1.52 (−11.1)	−2.94 to −0.11	0.109
RML	9.57	7.34	−2.02 (−21.1)	−3.15 to −0.88	0.109
RLL	12.76	10.26	−1.39 (−10.9)	−3.71 to 0.93	0.285
RIB	14.63	12.42	−1.23 (−8.4)	−3.88 to 1.41	0.593
RUL B1	3.97	3.56	−0.42 (−10.6)	−0.70 to −0.14	0.109
RUL B2	3.31	3.88	0.39 (11.7)	−0.10 to 0.87	0.285
RUL B3	3.59	3.33	−0.02 (−0.7)	−0.39 to 0.34	0.593
RML B4	3.24	3.12	0.13 (4.1)	−0.12 to 0.38	0.285
RML B5	3.23	3.21	−0.07 (−2.2)	−0.31 to 0.17	0.593
RLL B6	3.75	3.19	−0.90 (−24.0)	−2.59 to 0.79	0.285
RLL B7	4.88	3.71	−1.27 (−26.1)	−2.33 to −0.22	0.109
RLL B8	4.06	3.12	−1.28 (−31.5)	−3.23 to 0.67	0.109
RLL B9	3.67	2.81	−0.66 (−18.0)	−1.13 to −0.19	0.109
RLL B10	3.63	2.96	−1.34 (−36.9)	−3.06 to 0.38	0.109

Abbreviations: CT, computed tomography; LLL, left lower lobe; LLL B6, superior segment; LLL B7/8, anterior medial basal segment; LLL B9, lateral basal segment; LLL B10, posterior basal segment; LMB, left main bronchus; LUL, left upper lobe; LUL B1/2, apicoposterior segment; LUL B3, anterior segment; LUL B4, superior lingular segment; LUL B5, inferior lingular segment; RIB, right intermediate bronchus; RLL, right lower lobe; RLL B6, superior segment; RLL B7, medial basal segment; RLL B8, anterior segment; RLL B9, lateral basal segment; RLL B10, posterior basal segment; RMB, right main bronchus; RML, right middle lobe; RML B4, lateral segment; RML B5, medial segment; RUL, right upper lobe; RUL B1, apical segment; RUL B2, posterior segment; RUL B3, anterior segment.

Negative values indicate a decrease, and positive values indicate an increase of the diameter.

**Table 3.** Length of Bronchial Segments From the Trachea Until the Segmental Level of Bronchi in Patients With Left and Right Pneumothorax.

Bronchus part	Median from inspirated CT scan, mm	Median from collapsed CT scan, mm	Length difference, mm (%)	Confidence interval	*P* value
Left pneumothorax					
LMB	58.07	59.15	−1.70 (−2.9)	−9.35 to 5.96	1.000
LUL	15.84	17.71	−1.06 (−6.7)	−4.67 to 2.55	0.593
LUL-B1/2/3	11.30	12.05	0.04 (0.4)	−2.25 to 2.33	1.000
LUL-lingula	17.54	12.32	−2.66 (−15.2)	−7.66 to 2.34	0.285
LLL-B6	16.57	16.59	−0.05 (−0.3)	−0.96 to 0.86	0.593
LLL-BB	15.04	15.85	−0.39 (−2.6)	−3.13 to 2.36	1.000
Right pneumothorax					
RMB	32.33	29.75	−5.51 (−17.0)	−12.73 to 1.71	0.109
RUL	16.46	16.89	0.48 (2.9)	−0.85 to 1.81	0.593
RML	20.46	18.94	−1.93 (−9.4)	−3.91 to 0.04	0.109
RLL-B6	9.04	7.19	−0.44 (−4.8)	−4.70 to 3.82	1.000
RLL-BB	19.77	19.95	0.03 (0.2)	−3.00 to 3.06	0.285
RIB	30.33	31.56	5.40 (17.8)	0.80 to 9.99	0.109

Abbreviations: BB, basal branches; CT, computed tomography; LLL, left lower lobe; LMB, left main bronchus; LUL, left upper lobe; RIB, right intermediate bronchus; RLL, right lower lobe; RMB, right main bronchus; RML, right middle lobe; RUL, right upper lobe.

Negative values indicate a decrease, and positive values indicate an increase of the length.

### Angular Differences

**Fig. 4.**
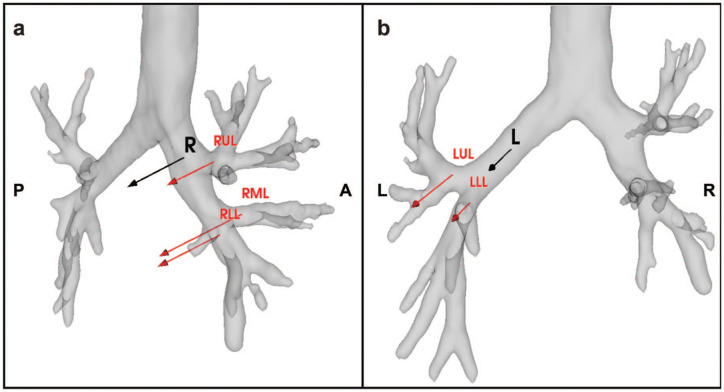
Vectors in the direction of the bronchial displacement during pulmonary collapse. The (a) right-sided and (b) left-sided median displacement vectors are represented by the black arrows. The median displacement vectors are shown in red separately per lobe for the right and left sides. The length of the arrow represents the size of the displacement, and the direction of the arrow represents the direction of the vector displacement (Table 4). The median bronchus displacements were 14.41 mm and 11.99 mm for the right-sided and left-sided bronchus, respectively. LLL, left lower lobe; LUL, left upper lobe; RLL, right lower lobe; RML, right middle lobe; RUL, right upper lobe.

In Figure 4, the median directions of bronchial displacement are shown for the right and left bronchial tree and per lobe. The median amount of displacement was 14.41 mm for a right-sided pneumothorax, mostly in the dorsomedial direction, and for the left-sided pneumothorax 11.99 mm, mostly in the dorsolateral direction (*P* = 0.79; Table 4).

**Table 4.** Angles of Bronchial Segments Until the Segmental Level of Human Lungs in Patients With Left and Right Pneumothorax.

	Axial X, °	Coronal Y, °	Sagittal Z, °	Displacement vector length, mm
Left-sided pneumothorax				
T-LMB	−11.55	−1.81	0.09	3.74
LMB-LUL	6.97	0.89	−21.02	5.04
LMB-LLL	−8.32	3.80	11.82	5.29
LUL-B1/2	43.10	−3.94	22.37	5.73
LUL-B3	11.63	−26.58	56.92	9.23
LUL-lingula	−1.43	4.04	34.26	4.54
Lingula-B4	19.97	12.59	48.46	7.61
Lingula-B5	−1.02	−3.58	−7.80	14.49
LLL-B6	10.92	18.14	10.93	6.31
LLL-BB	−19.66	−3.23	12.46	5.20
Right-sided pneumothorax				
T-RMB	8.20	−0.25	−2.33	7.10
RMB-RUL	−3.46	−27.08	−109.78	10.76
RMB-RIB	−2.69	3.69	−6.27	11.97
RIB-RML	−12.69	8.72	−2.49	18.53
RIB-RLL	0.62	−1.20	6.05	13.72
RUL-B1	−3.55	−6.11	49.55	13.58
RUL-B2	7.52	−0.27	−52.75	20.69
RUL-B3	−9.79	−44.38	13.83	13.43
RML-B4	−9.42	−3.90	9.30	20.84
RML-B5	3.38	26.28	8.82	19.55
RLL-B6	−3.60	13.73	−26.55	13.14
RLL-BB	−7.59	8.04	3.26	14.45

Abbreviations: LLL, left lower lobe; LLL B6, superior segment; LMB, left main bronchus; LUL, left upper lobe; LUL-B1/2, apicoposterior segment; LUL-B3, anterior segment; LUL-B4, superior lingular segment; LUL-B5, inferior lingular segment; RIB, right intermediate bronchus; RLL, right lower lobe; RLL-B6, superior segment; RMB, right main bronchus; RML, right middle lobe; RML-B4, lateral segment; RML-B5, medial segment; RUL, right upper lobe; RUL-B1, apical segment; RUL-B2, posterior segment; RUL-B3, anterior segment; T, trachea.

Positive angles indicate displacement in the counterclockwise direction around the respective axis, and negative angles indicating displacement in the clockwise direction around the respective axis.

### Algorithmically Modeled Centerline

**Fig. 5.**
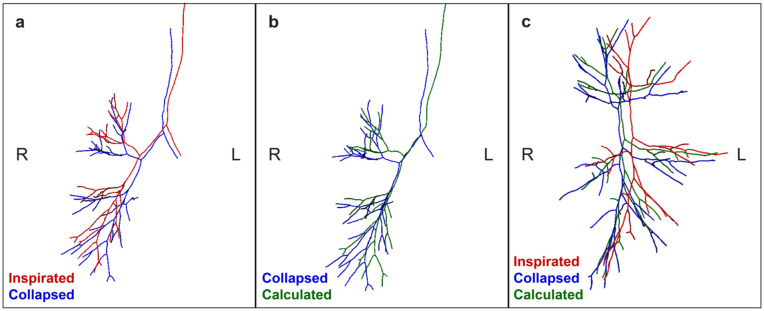
Bronchus centerlines of a patient in inspirated, collapsed, and algorithmically modeled pneumothorax states. (a) Frontal view of the inspirated (red) and collapsed (blue) bronchus centerlines. Because the pneumothorax is on the right side, the centerlines on the left side are largely similar on the left side. On the right side, the collapsed centerline is deformed more dorsomedially. (b) Frontal view of the collapsed (blue) and algorithmically calculated (green) bronchus centerlines of the same patient. The collapse deformations have been applied on the inspirated bronchus centerline resulting in the modeled bronchus centerline, which is approaching the position of the collapsed bronchus. (c) Lateral view of the inspirated, collapsed, and calculated bronchus centerlines. The calculated collapsed centerline is approaching the “real” collapsed bronchus.

Finally, we applied all calculated deformations, including angular displacement and length differences per patient case onto the inspirated centerline, resulting in an algorithmically modeled collapsed centerline. In Figure 5, an example is shown of the 3 different centerlines. The algorithmically modeled centerline is approaching the anatomy of the collapsed centerline.

## Discussion

We performed an algorithmic analysis calculating human and porcine lung deformations due to pneumothorax, per anatomical bronchial branch, based on real-world CT data, to develop a mathematical approach for future use in intraoperative AR guidance. The right-sided displacements were greater than the left-sided displacements; however, in the setting of small numbers of observations, no statistical significance was found. Nevertheless, this could be the result of the anatomical differences between left and right, such as the position of the heart and the size of the thoracic cavities. Moreover, it was noticeable that B4 and B5 (the lingula on the left side, and middle lobe on the right side) were displaced more than the other segments of both lobes. This could be due to their size, an appended relation to the adjacent lobe(s), and being more mobile, allowing for more displacement. Volume differences were comparable to other studies in the literature for human and animal pneumothorax models.^8,14^ We were able to apply the calculated deformations on the inspirated bronchus centerline, which resulted in an artificially modeled collapsed centerline. In the future, these deformations could also be applied for preoperative patients to predict the intraoperative bronchial collapse using the bronchus centerline of the preoperative CT scan. The clinical implication of predicting this spatiotemporal displacement of the lung is particularly relevant in detecting small noduli not superficially located, for example, in the case of metastasectomy or diagnostic (endobronchial) procedures. Another clinical application of these models could be the prediction of lung structure displacement in the preoperative planning of anatomical lung resections such as lobectomy and segmentectomy. These models could be used for intraoperative navigation during complex anatomical resections such as segmentectomy procedures. We expect that these models correlate with the intraoperative anatomy better than the contemporary preoperative inflated models (CT-based reconstruction techniques or 3D-printed models). This could facilitate better intraoperative simulation of the distorted anatomy and potentially improve tumor localization, leading to better surgeon performance, increased operation flow, and finally to fewer complications.

Although the results of the current study are promising for the development of intraoperative AR-guided navigation models, this study has several limitations that need to be mentioned. First, we were able to include only 2 sets of porcine lungs and 6 patients, of whom 3 had a right-sided and 3 had a left-sided total pneumothorax. Including more patients would improve the accuracy of the algorithmic model. Unfortunately, we screened more than 350 patients and found only 6 eligible cases with a total pneumothorax, with at least 2 available CT scans of a total pneumothorax and inflated lung. We did not want to perform the missing follow-up scans to prevent additional unnecessary radiation exposure for the patients.

Second, another limitation is the difference in positioning: for the porcine lungs, because of the ex vivo setting, missing the chest wall and thoracic cavity, leading to loss of physiological intrathoracic pressures and resistance, resulting in an “unnatural” collapse. Unfortunately, due to regulations and technical limitations, we were not able to perform full-body porcine CT scans. Moreover, the patients were in the supine position during the CT scans, but intraoperatively during pulmonary surgery, they are usually in lateral decubitus position. This altered positioning should be accounted for in the deflation algorithm.^15^

Third, we included only the bronchial displacements and the volume differences in our deformation algorithm. Including the parenchymal deformations is important for intraoperative guidance and tumor localization, as the lung parenchyma not only is reduced in volume but also shows deformations.^6,8^ We suppose the arterial deformations to be comparable with the bronchial deformations, given their close embryological relationship and connection in the pulmonary hilum. For the venous deformations, this assumption may hold within the hilum, because of their proximity and connection; however, in further peripheral regions, this relationship is less certain and requires further validation. We have yet to implement the vascular and parenchymal deformations in the model, and further validation is needed to determine whether (or to what extent) the observed bronchial displacements can reliably be extrapolated to the arterial and venous vasculature. In addition, it is also important to further substantiate the accuracy of the collapsed reconstructions, for example, by minimizing the centerline distance.

Furthermore, in this study, the calculations were based on pneumothorax lungs of otherwise pulmonary healthy patients, but the appearance of a large tumor, post–obstructive atelectasis, or emphysema could influence how the lungs deflate and would alter the results of the current calculated deformations. Finally, intraoperatively, the degree of collapse potentially differs from a clinical pneumothorax, and the collapse (due to atelectasis) increases as the surgery proceeds; this might also increase the deformation factor. In addition, using carbon dioxide inflation intraoperatively in the thoracic cavity (in some cases) can also increase the collapse and atelectasis, resulting in increased deformation as well. To overcome these limitations, intraoperative imaging data such as CBCT may be promising, as it is the actual intraoperative deflated condition of the lung we aimed to replicate in lateral decubitus positioning. Besides the intraoperative altered position, the effect of different pathologies could also be studied and compared with our current outcomes regarding otherwise healthy lungs. Nonetheless, CBCT is not widely used in pulmonary surgery due to the need for specialized equipment and the higher radiation exposure.^16^ To strengthen our results, it is necessary to expand this dataset, and simultaneously also to include pathologies and intraoperative imaging data. This could be achieved through collaboration with multiple international centers. Federated learning could facilitate secure collaborations, enabling collaboration and training models, without exchanging raw patient data.^17^

The next step will be generating an artificially collapsed bronchus for a patient selected for pulmonary anatomical resection. This collapsed bronchus model could then be used as input for a 3D-AR overlay.^2^ Next, intraoperative validation of the results needs to be performed, by comparing the artificially collapsed 3D-AR model with the intraoperatively collapsed bronchus to assess the accuracy and applicability of the calculated deformations. Future research should focus on a full 3D reconstruction including pulmonary vasculature as well as parenchyma.

## Conclusions

Algorithmically driven calculations were performed of lung deformation after the collapse of human and porcine lungs. This method could be a foundation for a dynamic deflation model, suitable for intraoperative AR-based pulmonary navigation. Intraoperative validation is essential to increase the accuracy of the model.
